# Assessing spatiotemporal population density dynamics from 2000 to 2020 in megacities using urban and rural morphologies

**DOI:** 10.1038/s41598-024-63311-5

**Published:** 2024-06-19

**Authors:** Jing Xie, Nan Wei, Quan Gao

**Affiliations:** 1 School of Geography and Planning, Sun Yat-Sen University, Guangzhou, China; 2 Southern Marine Science and Engineering Guangdong Laboratory (Zhuhai), Zhuhai, China

**Keywords:** Population, Urbanization, Local climate zones, Urban–rural morphology, WorldPop, Greater Bay Area, Socioeconomic scenarios, Environmental impact

## Abstract

Rapid urbanization has resulted in the substantial population growth in metropolitan areas. However, existing research on population change of the cities predominantly draws on grid statistical data at the administrative level, overlooking the intra-urban variegation of population change. Particularly, there is a lack of attention given to the spatio-temporal change of population across different urban forms and functions. This paper therefore fills in the lacuna by clarifying the spatio-temporal characteristics of population growth in the Guangdong-Hong Kong-Macao Greater Bay Area (GBA) from 2000 to 2020 through the methods of local climate zone (LCZ) scheme and urban–rural gradients. The results showed that: (1) High population density was observed in the compact high-rise (LCZ 1) areas, with a noticeable decline along urban–rural gradients. (2) The city centers of GBA experienced the most significant population growth, while certain urban fringes and rural areas witnessed significant population shrinkage. (3) The rate of growth tended to slow down after 2010, but the uneven development of population-based urbanization was also noticeable, as urbanization and industrialization varied across different LCZ types and cities in GBA. This paper therefore contributes to a deeper understanding of population change and urbanization by clarifying their spatio-temporal contingences at landscape level.

## Introduction

Population density and its variation are topics of continuous interest in urban, environmental, and human geography research. The accurate evaluation of the spatial–temporal distribution and change of urban population density can help us better discern the spatial processes of urban sprawl or shrinking^1^, land use change^2^, diffusion of epidemics^3^, industrial and commercial activities^4^, and urban eco-environments^5^. Understanding the patterns and evolution of population density and distribution is therefore essential for urban planning and socio-economic development.

Some early research of urban economics view population density as primarily an issue related to a function of the distance to central business district (CBD) of a city^6^, yet this may overlook the variegation of urban forms and the influence of transport and technological change that bring to more decentralized or multi-centered patterns of population distribution^7^. Other scholars link population density to the informal settlements (such as slum and China’s “urban village”)^8^, energy intensity^9^ and the inflows of internal migration into the city^10^. In terms of the temporal change of population density, a line of research has found that the average urban population densities have declined in the past several decades in most cities across the world^11,12^, mainly owing to the outweigh of the rate of urban built environment expansion over that of population growth^13,14^. This general trend is also noticeable in China despite the massive urbanization in the past four decades^15^

Notwithstanding scholars’ continued interests in urban population density, little attention has been given to the population change across the spatial variation within cities and especially to the coupling relation between population density and urban layout^16^. To associate population density with urban form and functions can help researchers and practitioners more accurately evaluate the ways and patterns that population varies across different size, scale and shape of built environments within the cities^17^. Essentially, some studies have endeavored to use various data sources such as census data, satellite imagery, and geospatial data to delineate the population and land use extents of cities^18–20^. For instance, Tian et al.^21^ used the land cover data to develop a China’s population distribution model (CPDM) that addresses the spatial pattern of population density of 1 km^2^ grid-cells. Other research shows that population density change is a crucial driving force of land use changes^22^ and land surface temperature^23^. However, these studies have not captured the internal variegations within urban landscape, necessitating the supplementation of landscape methodology to more clearly convey the physical properties, forms and shapes of urban surface, especially the internal structure and functions of cities.

The local climate zone (LCZ) is a suitable classification system to address urban surface in a holistic base^24^. LCZ has been used as a description of urban land into recognizable types with “uniform surface cover, structure, material, and human activity that span hundreds of meters to several kilometers in horizontal scale”^24^. LCZ is composed of 10 built types (see Table 1) that are associated with particular urban canopy parameters and 7 land cover types (see Table S1), each of which has coherent attributes that can distinguish with one another. Although it is originally designed for investigating effects of the urban temperature and urban heat island across different built environments, the LCZ scheme has been adopted into a wide range of application such as urban planning^25^ and urban health issues^26^. However, there is limited research that has paid attention to the population distribution across the spatial–temporal difference of LCZ types, except a few studies trying to involve the variables of urban properties into the mapping of population distribution^16,27^. For example, drawing on the dataset from 34 cities in China, Zhou et al.^27^ compare the population density of local climate zones (LCZs) in cities of different scales. Demuzere et al.’s^16^ research use LCZs approach to examine population density across different physical characteristics of the cities in the continental US such as building footprints, height and surface area.

**Table 1:** Built types of local climate zone (LCZ) classification.

Example	Built types	Example	Land cover types
	LCZ 1: Compact high-rise		LCZ 6: Open low-rise
	LCZ 2: Compact midrise		LCZ 7: Lightweight low-rise
	LCZ 3: Compact low-rise		LCZ 8: Large low-rise
	LCZ 4: Open high-rise		LCZ 9: Sparsely built
	LCZ 5: Open midrise		LCZ 10: Heavy industry

Nevertheless, to more comprehensively evaluate the patterns of population density in different LCZ types, we need to further consider at least three issues that existing literature has not fully addressed. First, the time series data needs to be introduced to fully capture the temporal evolution of population distribution across LCZ types within the cities. As existing research has documented the high-speed of urbanization in China and especially in the GBA in the past two or three decades^28^. A LCZ approach therefore can help enrich the understanding of urbanization in GBA by considering the more detailed intra–urban changes of population and LCZ types. Second, despite the recognition of internal characteristics of urban form, most research have not explored the urban–rural gradients of both population growth and LCZs change. Delving into the urban–rural gradients thus can advance our understanding of the variegated urbanization processes by identifying the growing and shrinking areas of the cities^29^ and how they manifest in the change of LCZs. Third, some LCZ types that characterize the urban conditions of developing countries deserve more in-depth exploration. For example, LCZ 7 (lightweight low-rise) often refers to informal settlements that may manifest as slums in India and Africa but hardly could be found at US. This paper therefore fills in the above-mentioned lacunas by evaluating the spatiotemporal growth of population (2000–2020) across urban–rural morphology gradients and LCZ types of 11 cities in the Guangdong-Hong Kong-Macao Greater Bay Area, China.

In this study, LCZ was used to simulate the urban–rural gradient and evaluate the temporal and spatial characteristics of population change in the GBA. The objectives are to reveal the population distribution of the GBA and 11 cities, and identify whether these cities are growing or contracting. This study will demonstrate the validity of LCZ for studying the gradient distribution of population along urban–rural areas. Our research will fill in the current gaps of using LCZ to simulate the urban–rural gradient to assess the temporal and spatial changes in population and will effectively distinguish the coupling relationship between population density and urban layout. Our research will be also helpful in understanding the internal spatial structure of megacities and in promoting a better grasp of the urbanization process. Our research aims to: (i) analyze the spatio-temporal distribution and variation characteristics of population along the urban–rural gradient in the overall GBA and its 11 cities from 2000 to 2020; (ii) identify the growth or contraction patterns within the GBA and its 11 constituent cities during the study period; (iii) analyze the population distribution changes along the urban–rural gradient within metropolitan areas by further examining spatial structural changes.

## Material and methods

### Study area

**Figure 1:**
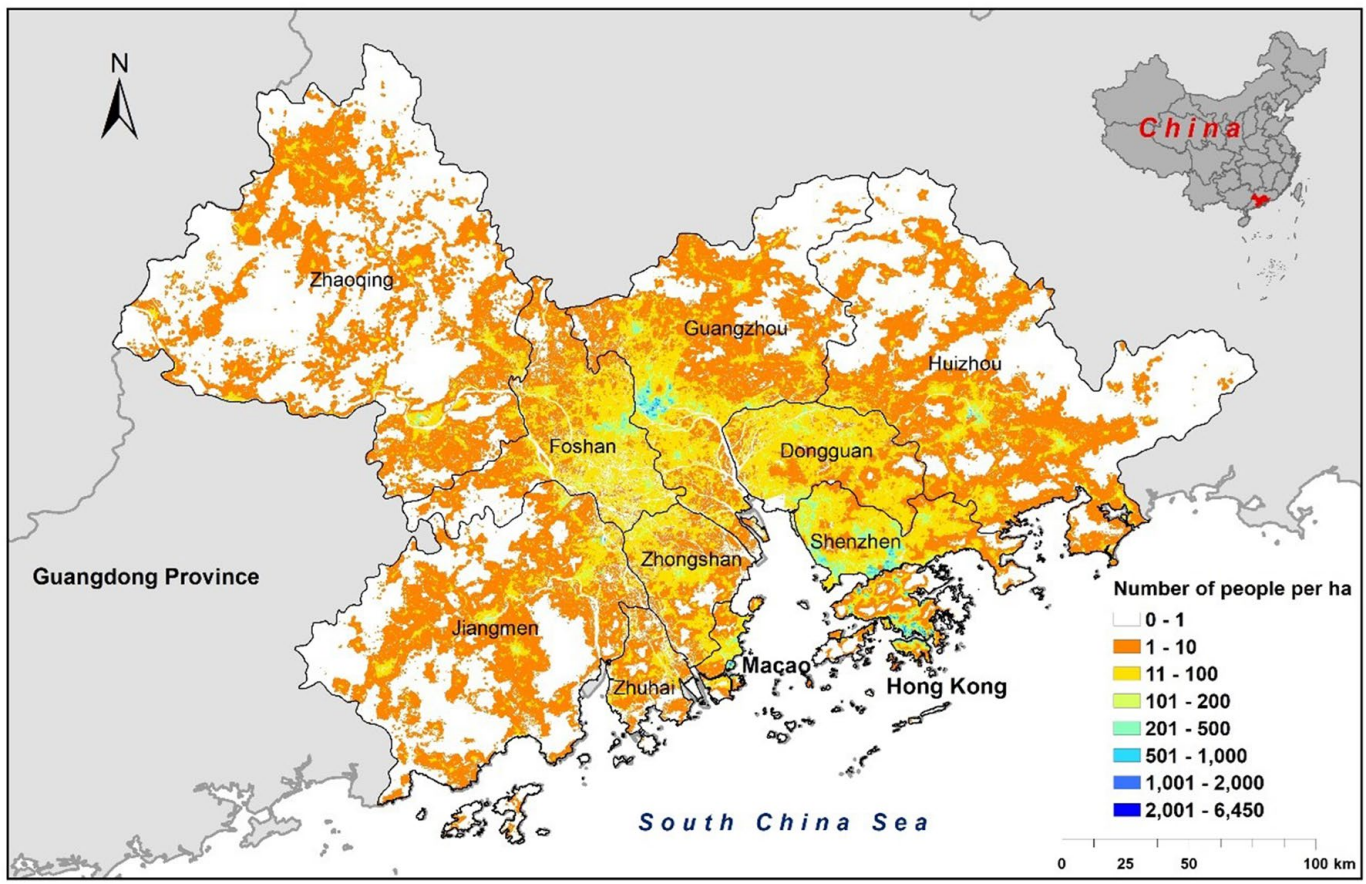
The spatial distribution of population in 2020 with cities in the Guangdong-Hong Kong-Macao Greater Bay Area (GBA). Population estimation obtained from WorldPop (http://www.worldpop.org). Figure mapping visualizations were performed in ArcGIS (v10.8, ESRI, USA).

The Guangdong-Hong Kong-Macao Greater Bay Area (GBA) consists of Guangzhou, Zhaoqing, Foshan, Huizhou, Jiangmen, Zhongshan, Dongguan, the two Special Economic Zones (SEZ) of Shenzhen and Zhuhai in Guangdong Province, the Macao Special Administrative Region (Macao SAR), and the Hong Kong Special Administrative Region (Hong Kong SAR). It is located on the southeast coast of China, in a subtropical climate region, with extensive low-lying plains near the Pearl River estuary and numerous mountains in the northern region (111.35–115.40°E, 21.56–24.40°N). As of 2020, the total area of the GBA is 55,900 km^2^, with a total population of approximately 86.17 million. The Guangdong-Hong Kong-Macao Greater Bay Area boasts superior geographical conditions, with mountains surrounding it on three sides and three converging rivers. It has a lengthy coastline, a well-developed port infrastructure, and a vast sea area. The economic hinterland is extensive, and the Pearl River Delta region accounts for about one-fifth of the national land area, one-third of the population, and one-third of the total economic output. As one of the most economically dynamic regions, the GBA plays an exceedingly significant role in promoting economic development in China^30–32^. Over the past few decades, as GBA has become one of the most important economic engines of China^33,34^, the GBA has undergone the drastic process of urbanization and industrialization. This has been accompanied by significant population growth and environmental changes^35–37^ (Fig. 1).

### Spatial distribution of population (2000–2020) in the GBA

Population data was obtained from WorldPop (http://www.worldpop.org—School of Geography and Environmental Science, University of Southampton; Department of Geography and Geosciences, University of Louisville; Departement de Geographie, Universite de Namur, accessed by December 2023) and the Center for International Earth Science Information Network (CIESIN), Columbia University (2018). This data is part of the Global High Resolution Population Denominators Project, funded by The Bill and Melinda Gates Foundation (OPP1134076) and can be accessed at 10.5258/SOTON/WP00660. The population dataset was available for download in Geotiff format at a resolution of 3 arc (approximately 100 m at the equator) and was used to estimate the total number of people per grid-cell. The projection used is the Geographic Coordinate System, WGS84, and the units are the number of people per pixel, with country totals adjusted to match the corresponding official United Nations population estimates. These estimates are provided by the Population Division of the Department of Economic and Social Affairs of the United Nations Secretariat (2019 Revision of World Population Prospects). The mapping approach utilizes Random Forest-based dasymetric redistribution to clarify the population distribution in the GBA region. The spatial distribution of the population in 2020 has been adjusted to align with the corresponding UNPD estimates for the total population of Macao and Hong Kong.

### Local climate zones and their urban–rural gradients

This study used LCZ maps from three time points: 1999, 2009, and 2019, as created by Xie et al.^33^. These maps partitioned the GBA into LCZ 1–10, representing different types of surface building coverage based on height and density. The overall accuracies of the three LCZ maps were 91.6%, 76.0%, and 93%, respectively, with corresponding Kappa values of 0.638, 0.566, and 0.864 (for detailed accuracy information, refer to Tables S2, S3 and Fig. S1 in Xie et al.^33^). When creating these LCZ maps, the geographic coordinate system was converted to the World Geodetic System 84 (WGS-84) and the Universal Transverse Mercator projection zone 49 North (UTM 49N). In this study, the nearest neighbor method was employed within the ENVI/IDL environment (v5.3, EXELIS Inc., McLean, VA, USA) to resample the LCZ maps to 100 m for subsequent spatial analysis. Surface coverings do not significantly change over short periods; thus, the LCZ types remained relatively consistent in the same regions between adjacent years. Therefore, conducting spatial analysis of population distribution for 2000, 2010, and 2020 based on LCZ maps from 1999, 2009, and 2019, respectively, is deemed feasible.

The urban–rural gradient was delineated to capture the continuous changes in urban–rural landscapes within the study area^38^. LCZ 1 (compact high-rise) areas typically exhibit the highest imperviousness and the lowest fraction of pervious surface^24^. In the GBA, LCZ 1 areas are located at the downtown zones and centers of urban domains^33,34^. Additionally, LCZ maps from 1999, 2009, and 2019 show that the locations of LCZ 1 areas have not undergone significant changes. Therefore, we chose the LCZ 1 areas from 2019 as the center to establish an urban–rural gradient covering the GBA. This approach accurately identifies multiple city centers, aligning with the current development status of the GBA. Based on the spatial pattern of LCZ 1 areas in 2019, the surveyed GBA exhibits a clear multi-center structure, although some metropolitan areas (such as cities in Europe and the United States) still display a single-center structure^39–41^. The specific method for establishing the urban–rural gradient involves calculating the Euclidean distance from each pixel to the LCZ 1 area and assigning pixels to different gradients based on distances in 250-m increments. The resulting gradient, with intervals of 250 m centered around the LCZ 1 area, has proven effective in characterizing urban–rural changes in urban elements (see Fig. S2). This study conducted an analysis of spatial and inter-annual population changes in the GBA based on the urban–rural gradient.

### Statistical analysis

In order to quantify the interannual variation in population density (PD) in the GBA and 11 cities during the research period, we calculated the interannual differences of PD (ΔPD) at the pixel level, which is the difference (expressed in years) between the current observation year and the previous year, adapting the approach described from Xie et al.^42^. Using interannual differences in PD (ΔPD) to measure the interannual changes in impact of PD on the interannual dynamics of service level.

Linear regression was used to test the urban–rural change trend of PD variables at a 250 m gradient level (significance defined as *p* < 0.001). Linear regression was also used to examine the interannual trend of PD variables at the pixel level over the past 20 years (significance defined as *p* < 0.05). We combined 250 m scale maps of the statistical results with the 250 m scale distance gradients, cities, and LCZ maps. They were then analyzed across distance gradients in the GBA. The characteristic of each distance gradient is the mean of statistical regression outcomes of significance (defined as *p* < 0.05). To disentangle the temporal dynamics of population growth, the inter-annual stability of PD and ΔPD were quantified as the inverse coefficient of variation (*CV*^−1^, no unit) to measure changing stability. All statistical analyses were calculated by using R programming (version 3.4.2). All mapping visualizations were performed in ArcGIS (v10.8, ESRI, USA).

## Results

### Spatial distribution of population depending on local climate zones and urban–rural morphology gradients between 2000 and 2020

**Figure 2:**
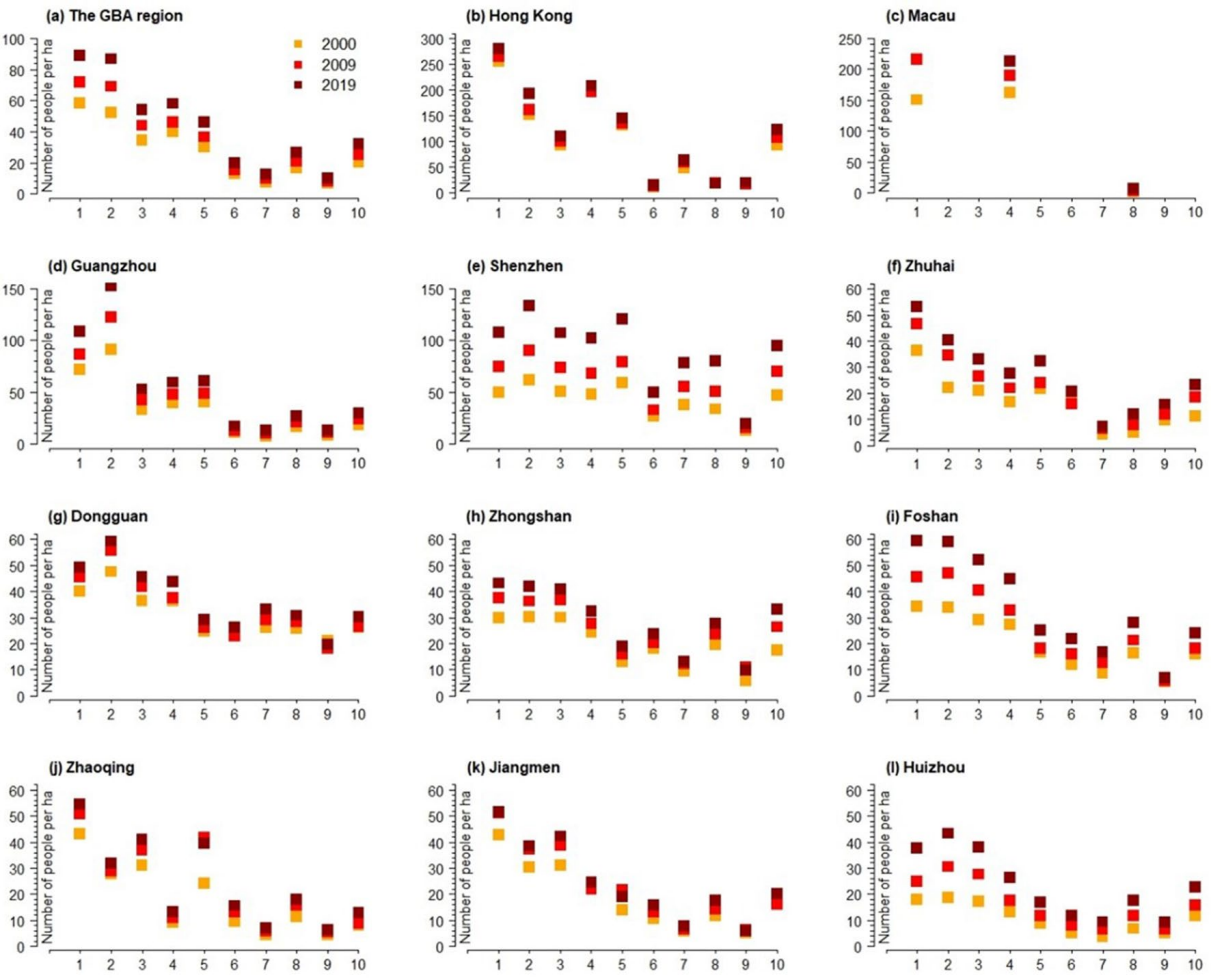
The spatial distribution density of population (number of people per ha) across the built LCZs (1–10) of 2000, 2009, and 2019 in GBA and the 11 cities.

The overall population distribution in the GBA is gradually decreasing along the urban–rural gradient (see Fig. 2). In general, compact high-rise (LCZ 1) areas, which are often associated with commodity housing, public housing and high-rise office building, accommodate the densest population in the GBA, averaging around 90 people per hectare. This particularly the case for cities like Hong Kong, Macau, Zhuhai, Zhaoqing and Jiangmen where highest population density locates in LCZ 1. Yet, in Guangzhou, Shenzhen and Dongguan, highest density of population mainly concentrates in LCZ 2 where compact mid-rise landed houses and “urban villages” are more likely to appear. This may be primarily due to the higher proportion of migrant population in these three cities, most of whom tend to inhabit informal settlements or “urban villages” with lower rent. For example, people without urban Hukou (China’s household registration system) constitute around 35%, 63% and 72% of the overall urban population of Guangzhou, Shenzhen and Dongguan respectively^43^.

**Figure 3:**
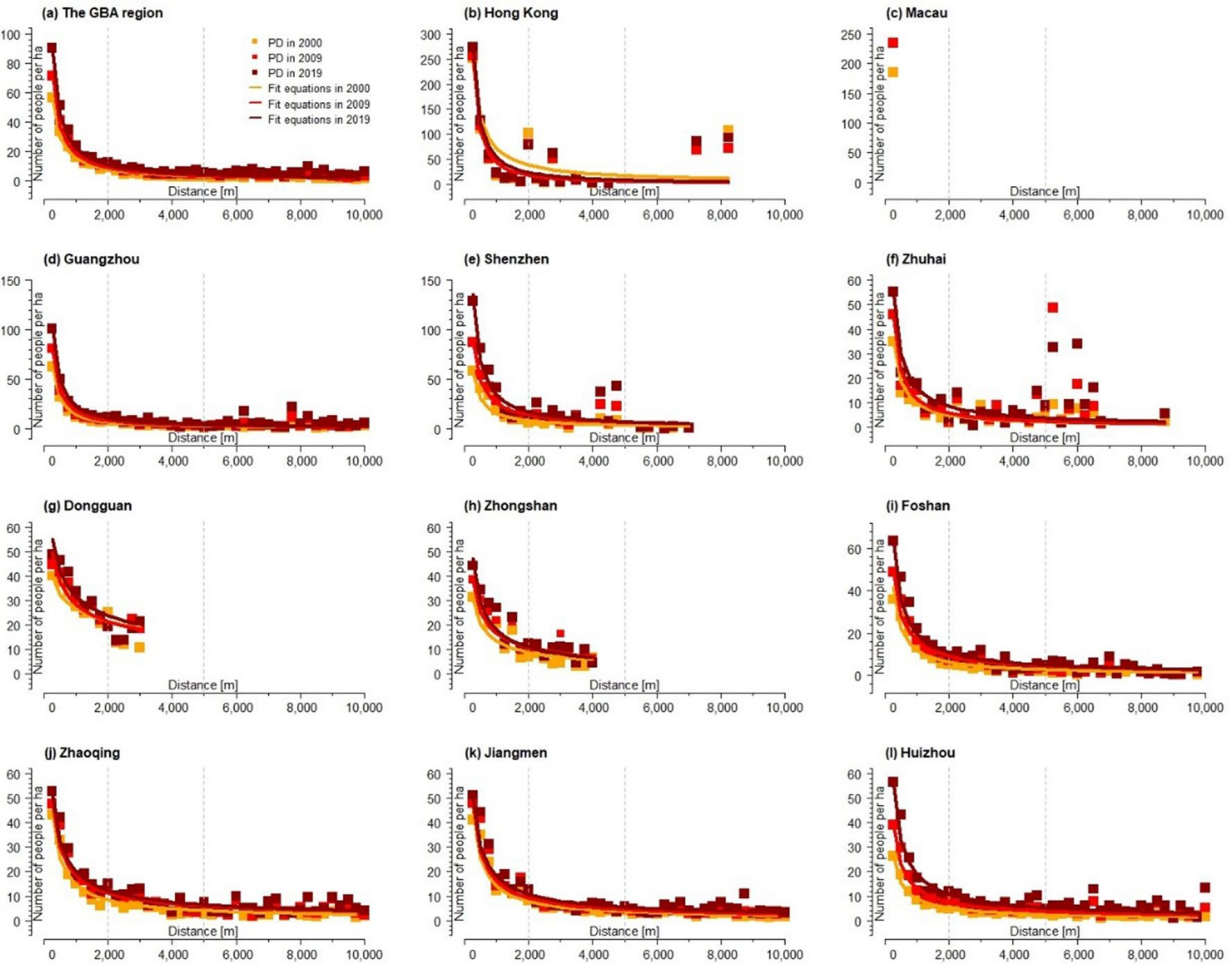
The spatial distribution density of population (number of people per ha) across the urban–rural gradients of 2000, 2009, and 2019 in GBA and the 11 cities.

In GBA, population density generally tends to decline across urban–rural morphology gradients (see Fig. 3), with average 90 people per hectare at the city center declining to around 10 people per hectare at the distance of 2000 m away from the city center. Moreover, within 2000 m of the city center, the curve drops sharply, indicating a rapid decline in population density along the urban–rural gradient within this range. Yet population density does not neatly decline along urban–rural morphology in each city. Hong Kong, Shenzhen and Zhuhai exhibit noticeable sub-centers with high population density. In particular, Zhuhai has two cores of high population density areas. We also use the linear regression to examine the urban–rural change of PD variables at a 250 m gradient level (see Table 2). The result shows that the cities of Guangzhou, Shenzhen, Foshan, Zhaoqing, Jiangmen and Huizhou show a positive correlation between population density and the distance to urban center.

**Table 2:** The fitting equations and precision of the spatial distribution density of population (number of people per ha) across the urban–rural gradients of 2000, 2009, and 2019 in the GBA and eleven cities.

Regions	2000	2009	2019
GBA	y = 11,000x^(−0.95)^,***	y = 18,500x^(−1)^,***	y = 22,500x^(−1)^,***
Hong Kong	y = 35,500x^(−0.9)^	y = 340,000x^(−1.3)^	y = 210,000x^(−1.2)^
Macau	–	–	–
Guangzhou	y = 28,000x^(−1.1)^,**	y = 35,000x^(−1.1)^,**	y = 45,000x^(−1.1)^,**
Shenzhen	y = 11,000x^(−0.95)^,*	y = 13,000x^(−0.9)^,*	y = 34,000x^(−1)^,*
Zhuhai	y = 6850x^(−0.95)^	y = 11,500x^(−1)^	y = 8000x^(−0.9)^
Dongguan	y = 210x^(−0.3)^	y = 450x^(−0.4)^	y = 500x^(−0.4)^
Zhongshan	y = 850x^(−0.6)^	y = 1050x^(−0.6)^	y = 2250x^(−0.7)^
Foshan	y = 6000x^(−0.9)^,*	y = 4100x^(−0.8)^,*	y = 7000x^(−0.85)^,*
Zhaoqing	y = 3650x^(−0.8)^,*	y = 2300x^(−0.7)^,*	y = 2500x^(−0.7)^,*
Jiangmen	y = 5000x^(−0.85)^,**	y = 4000x^(−0.8)^,**	y = 3200x^(−0.75)^,**
Huizhou	y = 2200x^(−0.8)^,*	y = 3300x^(−0.8)^,*	y = 4700x^(−0.8)^,*

A “y” is density of population (number of people per ha) and a “x” is the distance of urban–rural gradients.

Fitting accuracy: ***denotes a p-value less than 0.001, **denotes a p-value less than 0.01, *denotes a p-value less than 0.05.

### Temporal variation of population depending on local climate zones and urban–rural morphology gradients between 2001 and 2020

**Figure 4:**
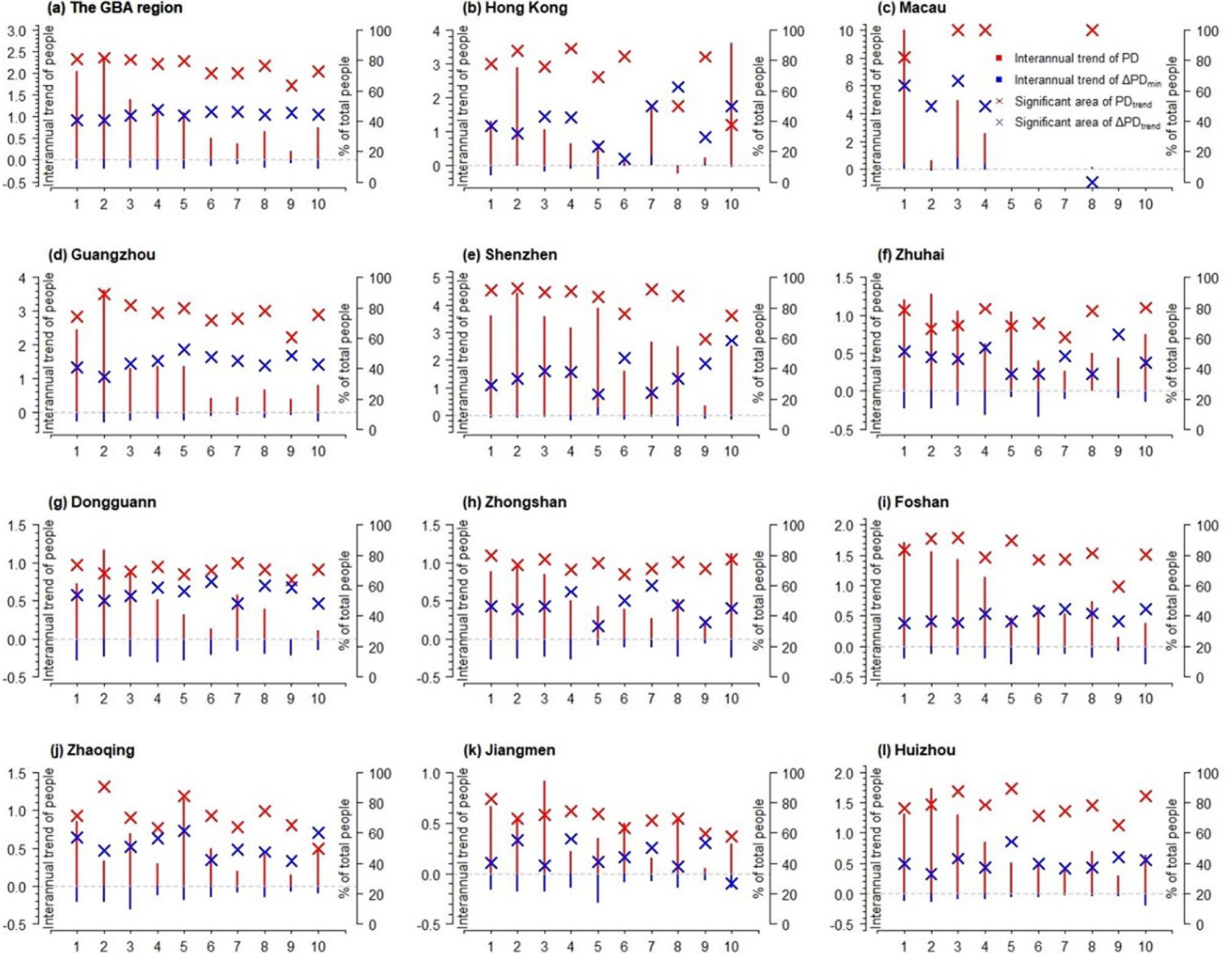
The interannual trend of amount and interannual difference (interannual trend of PD and interannual trend of ΔPD, respectively) on the density of population (number of people per ha) and the corresponding areas on significant trend (significant trend of PD and significant trend of ΔPD, respectively) across the built LCZs (1–10) between 2000–2020 in GBA and the 11 cities.

The interannual variation trend of population density shows a downward trend along the urban–rural gradient, and the downward trend is relatively obvious, indicating that there was significant population growth in all regions of the GBA during the study period, which are significant in above 40% of the total population (see Fig. 4). However, the peak values of interannual trend changes in population are mostly concentrated in LCZ 1–4 areas, indicating a significant increase in population density in densely populated urban areas. Compared to other regional types, the population growth is faster in densely populated urban areas with almost constant area. In cities of Guangzhou, Shenzhen, Zhuhai and Dongguan, LCZ 2 has experienced the most drastic growth in population, which may be primarily due to the inflow of large number of migrant workers who tend to live in the compact mild-rise housing and “urban villages”. In Macao, as urban built environments were highly compact already in 2001, LCZ 1 has become the dominant area for accommodating new growth population during 2001–2020. In Hong Kong, while urban center (LCZ 1 and 2) experienced noticeable population growth, the much larger proportion of population growth occurs at LCZ 10 which locates at the city’s port industrial areas (see the Fig. S3 of the supplementary materials). This was primarily attributed to the regeneration of some port areas into residential areas in the past two decades. The significant population growth is also noticeable in the LCZ 10 of Shenzhen, Zhuhai and Zhongshan where manufacturing factories concentrate. The factory dormitories had become a major source of housing for accommodating a large number of rural–urban migrant workers in these cities (Pun 2016).

**Figure 5:**
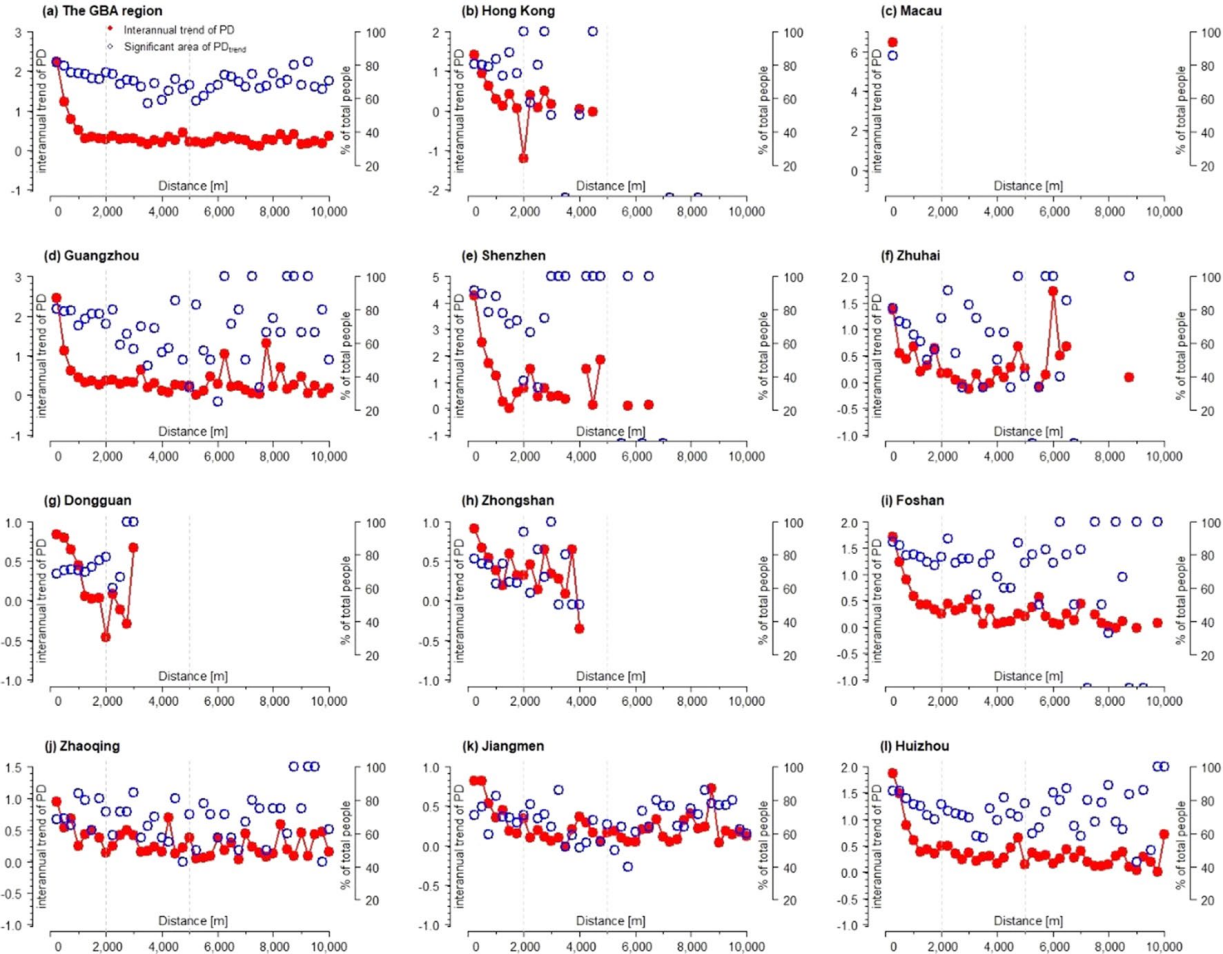
The interannual trend of the density of population (number of people per ha) and the corresponding areas on significant trend (significant trend of PD_trend_) between 2000–2020 across the urban–rural gradients in GBA and the 11 cities.

From a perspective of the urban–rural gradient, the city centers of GBA experienced the most significant growth of population, while some urban fringes and rural areas bore significant shrinkage (see Fig. 5) during 2000 to 2020. In Hong Kong, population density at the distance of 2000 m away from city center had averagely reduced 1% per year during 2000 to 2020, which made the population growth more concentrated within the metropolitan area. The cities of Zhongshan and Foshan also experience significant shrinkage at urban fringes, which means urbanization in the past two decades or so does not necessarily lead to population growth at the suburbs of these cities. For Zhuhai, it had developed the second polar of population growth at the distance of around 5000 m and 3000 m away from the old city centers, which results in the “U” shape of population growth along the urban–rural gradient. As the two city centers of population growth in Zhuhai had increasingly polarized during 2000–2020, the areas between the two city centers (at the distance between 3000 and 4000 m in Zhuhai) witness noticeable population shrinkage. In other words, population shrinkage does not necessarily take place in the urban fringes but sometimes occurs in the areas between two city centers. This “U” shape pattern of population growth across urban–rural gradients is also evident in Donguan, where new center of population growth occurs at the distance of around 3000 m away from old urban center and population shrinkage occurs at the area between the two urban centers.

**Figure 6:**
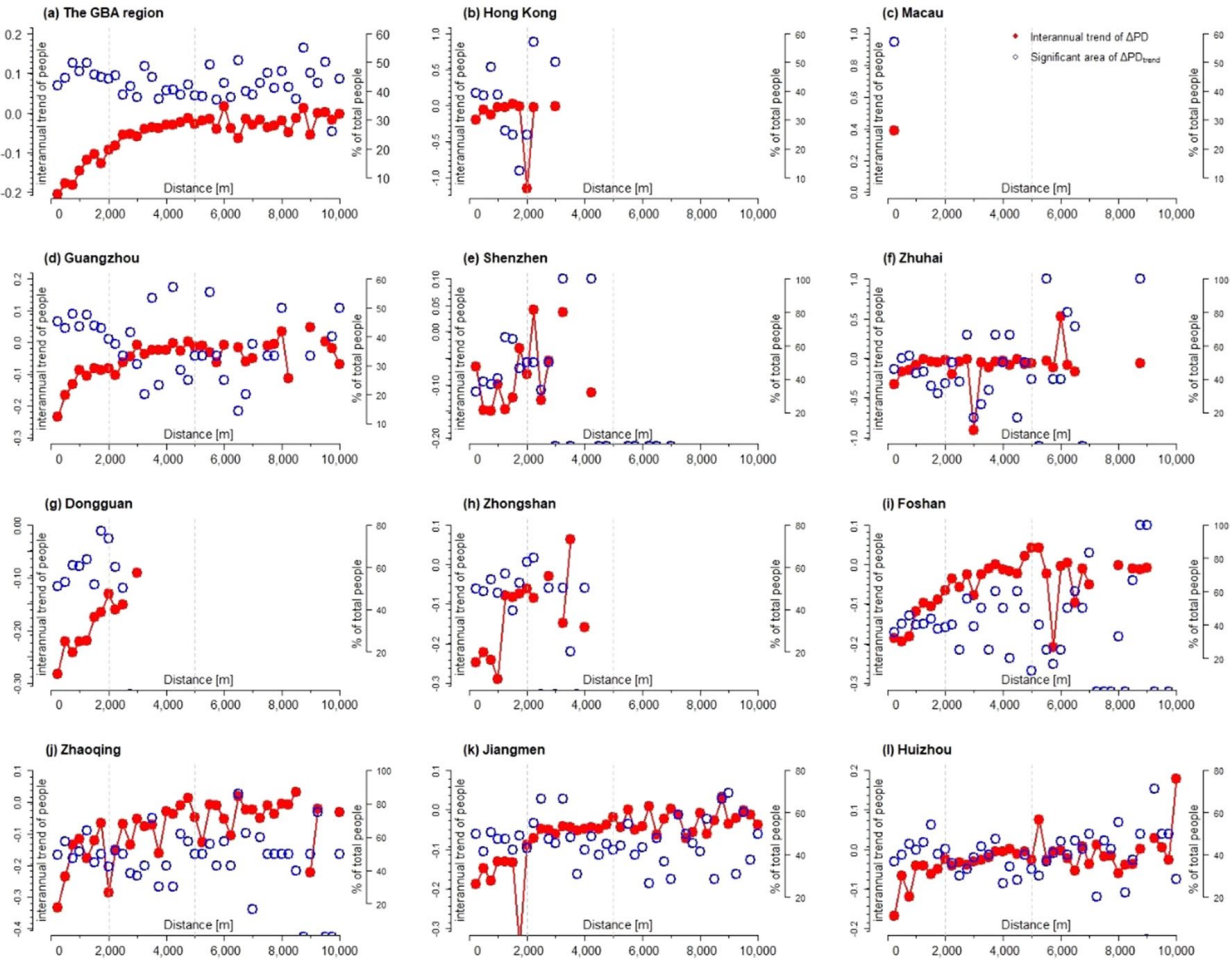
The interannual trend of the interannual difference of population (number of people per ha) and the corresponding areas on significant trend (significant trend of ΔPD_trend_) between 2000 and 2020 across the urban–rural gradients in GBA and the 11 cities.

In term of the rangeability of population growth, although the cities in GBA region experience population growth in most areas, the range of growth in average tends to decline during 2000 to 2020 (see Figs. 6 and S4). This is particularly the case in cities of Guangzhou, Shenzhen, Dongguan, Zhongshan, Foshan, Zhaoqing, Jiangmen and Huizhou, where the decline of population growth at central areas is the most significant.

### Key changes of spatial and temporal population changes between 2001 and 2020

**Figure 7:**
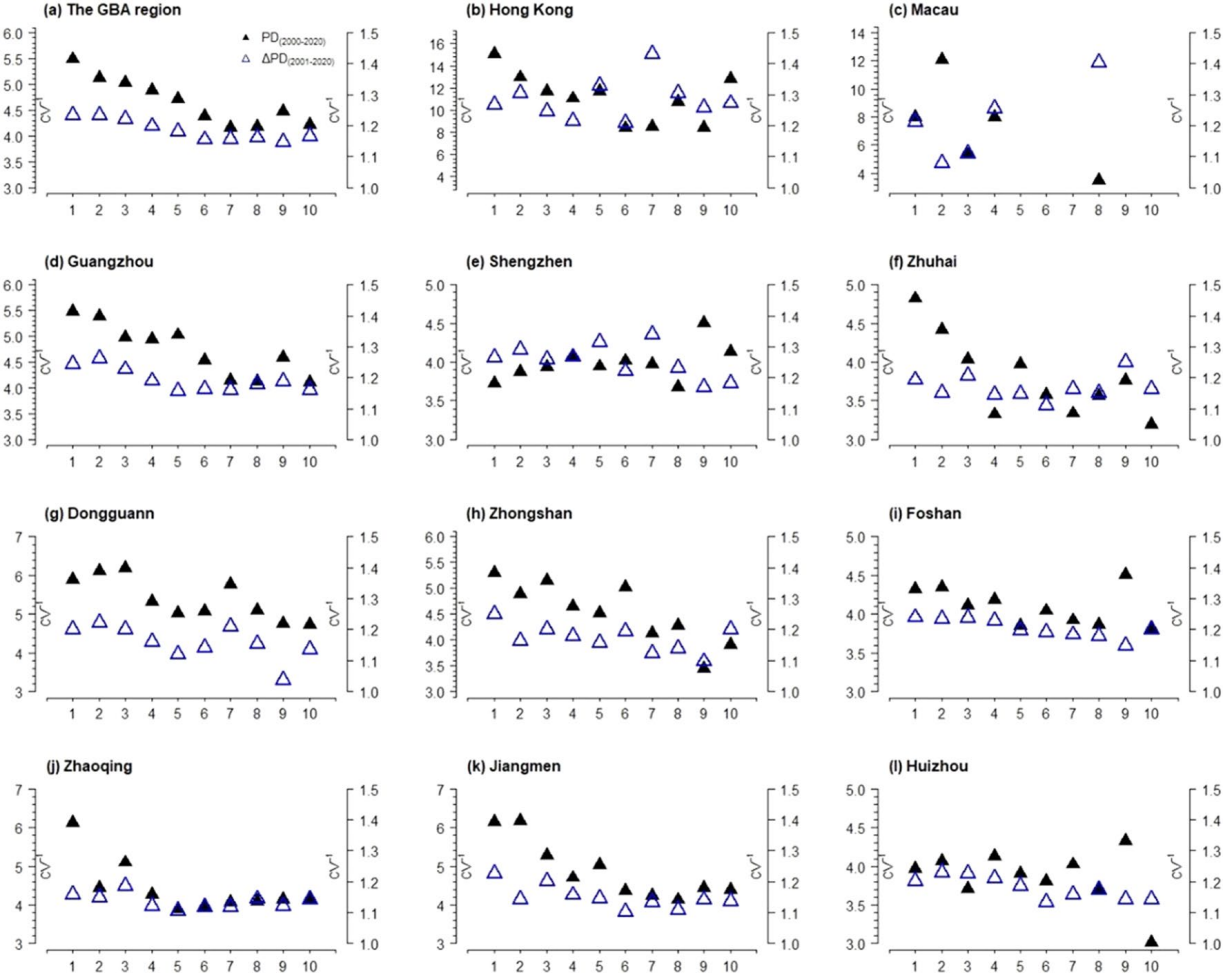
Inverse coefficient of variation (*CV*^-1^) of interannual value and abstract interannual difference (Δ) on the density of population (number of people per ha) (PD (2000–2020) and year of ΔPD (2001–2020), respectively) across the built LCZs (1–10) between 2000–2020 in GBA and the 11 cities.

In the GBA, the fluctuation range of the inverse coefficient of variation of PD and ΔPD was large (see Fig. 7), which means that the absolute and annal growth of population density are both consistent and stable during 2001 to 2020. In other words, the speed of growth of population density tends to slow down. In general, the population growth within the compact built environments (LCZ 1–3) tend to be more stable than that of open or mid-rise areas (LCZ 5–10). In particular, the most noticeable population growth measured by range of change was observed in LCZ 7 (lightweight low-rise), LCZ 8 (large low-rise), LCZ 9 (sparsely built) and LCZ 10 (heavy industry), which means some suburb areas of the cities had been gradually exploited during 2001–2020. However, this general pattern varied across different cities (see Figs. S5 and S6). In Shenzhen, the most fluctuated and noticeable population change was observed in the LCZ 1, LCZ 2 and LCZ 3 (the central and residential areas of the city), in which the largest amount of inflowing population was received (see also Fig. 2). If we compare Fig. 7 with Fig. 2, we also find that LCZ 9 in Shenzhen, Foshan, Huizhou were largely left unexploited during 2001 to 2020, as no noticeable population change was observed.

**Figure 8:**
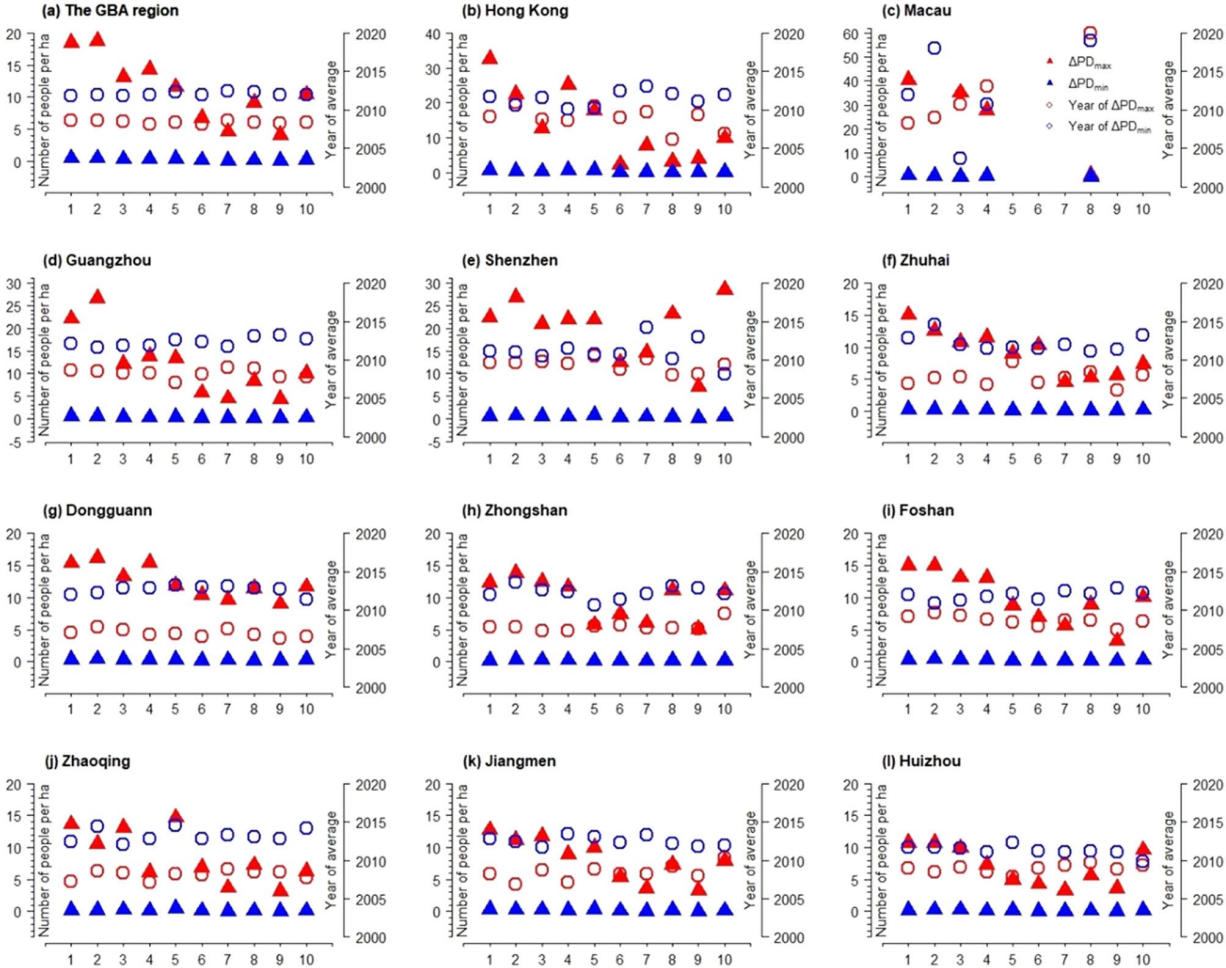
The maximum and minimum values of the interannual difference (ΔPD_max_ and ΔPD_min_, respectively) on the density of population (number of people per ha) and the corresponding years (year of ΔPD_max_ and year of ΔPD_min_, respectively) across the built LCZs (1–10) between 2000 and 2020 in GBA and the 11 cities.

Figure 8 shows the ΔPD_max_ and ΔPD_min_ along with their corresponding years, which provides further insight into the intercity variation characteristics of the population across different LCZs (see Fig. S7). The minimum value of the interannual variation difference in population was 0, which mainly occurred after 2010 and was concentrated between 2010 and 2015 across all LCZs. The maximum value of the interannual variation difference in population showed significant variations, which primarily occurred before 2010 and concentrated between 2010 and 2015. Moreover, the maximum interannual variation difference often occurred in LCZ 1 and LCZ 2, which took place between 2005 to 2010. In the words, the population density growth within the central areas of the cities reached the peak period during 2005–2010.

## Discussion

### Spatial distribution of population depending on local climate zones and urban–rural gradients between 2001 and 2020

At present, most of the methods used to map out population dynamic change are still based on the grid calculation and the statistics of population distribution. Drawing on data regarding the built area fraction per pixel or the population distribution per pixel value of the weighted population likelihood layer, this line of research tends to establish the estimated population distribution at the administrative level^18^. For example, land-use data and census data in a 1-km^2^ grid cell system are generated to assess urban growth and population density changes^1^.

Because the data based on grid scale division can only determine the population distribution and changes in a given urban or rural areas, it cannot accurately capture the internal variegation as well as the urban–rural gradients of an area. It is in this sense that we applied the new research method LCZ to simulate the rural–urban gradient, and analyzed the distribution and change of population in the Guangdong-Hong Kong-Macao Greater Bay Area (GBA) along the rural–urban gradient. Using population density as a measurement index, the spatial distribution of the GBA population in 2020 (see Fig. 1) had been charted. The research indicated that the population density distribution within the GBA was imbalanced, with noticeable population concentration areas including Tianhe District, Yuexiu District, Haizhu District, Liwan District, and the southwest of Baiyun District in Guangzhou, as well as in Shenzhen, Hong Kong, and other agglomeration regions. Furthermore, the population distribution along the Guangzhou-Dongguan-Shenzhen-Hong Kong axis was significant.

On the basis of defining the population agglomeration area in GBA, LCZ was applied to establish the urban–rural gradient to further reveal the population distribution along these gradients in GBA during 2001–2020. The study showed that the population density was concentrated in the area of compact high-rise (LCZ 1) areas. Moreover, the more compact and higher the buildings in the area were, the higher the population density in the area was (see Fig. 2). However, Guangzhou, Dongguan and Shenzhen were the exceptions, and the highest population density in these three cities was concentrated in the LCZ 2 and 3 area. In the past two decades or so, as rapid urbanization and industrialization took place in these three cities, they became the major cities accommodating rural–urban migrant workers in GBA area. Most of these migrant workers without urban properties and hukou predominantly concentrated on the self-built rental houses and the “urban villages” which normally coupled with LCZ 2 and 3. Our research also enriches the research that examines how internal migration may shape the population distribution across urban morphology^10^.

This paper also shows, by calculating Euclidean distances, that the population density in GBA was mainly distributed in urban areas and declines along the rural–urban gradient (see Fig. 3). However, Hong Kong, Shenzhen, and Zhuhai did not have a gradual decline in population along the rural–urban gradient, but rather had distinct sub-centers of high population density (see Fig. 3). This finding also confirms that the structure of these cities was multi-core structure^44^. In addition, Zhuhai had two obvious sub-centers with high population density, which may be due to distribution of the manufacturing industrial zones where a large number of migrant workers concentrated^45^.

### Spatial–temporal change of population density depending on local climate zones between 2001 and 2020

The temporal analysis on the population density change across urban–rural gradient shows that most areas of GBA experienced population growth, which is consistent with existing literature that GBA maintained sustained level of attraction for internal migrants during 1990 to 2020^46^. Yet, this paper provides a more comprehensive evaluation of the population change by calculating the inter-annual difference of population density at the pixel level so as to measure the inter-annual change in population density (see Fig. 7). We found that, although the population increased during 2001–2020, the inter-annual trend of population growth tended to slow down after 2010. In terms of the inter-annual differences in population density (see Fig. 8), the study found that the maximum and minimum values of inter-annual change in population density were concentrated between 2005–2010 and 2010–2015 respectively. This means that the most drastic urbanization reflected by population growth was taking placing during 2005–2010 in GBA in the past two decades. During this period, the GBA and especially the cities in Pear River Delta experienced the fastest development of export-oriented economy, which attracted the inflows of a large number of migrant workers in the industrial areas of the cities^47^. However, the export-oriented economy in GBA was affected by the 2008 global finical crisis and the large scale of spontaneous and state-sponsored industrialization and urbanization had tend to stabilize.

This also confirms, to a certain extent, that the urbanization in the GBA has entered the late stage, meaning that the population-based urbanization of GBA reached more than 70% in 2021 (http://stats.gd.gov.cn/). The analysis of population change based on urban morphologies and LCZ shows that city center and especially the LCZ 1–3 were still the key spaces for accommodating the absolute quantity of population growth, although the growth of population in suburban and industrial areas were more noticeable in terms of the annal fluctuation range. In particular, the heavy industrial areas (i.e., LCZ 10) show the smallest Inverse coefficient of variation (*CV*^-1^) in most cities of GBA. In other words, LCZ 10 undergone more intensive exploitation and inter-annual population growth than that of the city centers. In this sense, the research helps us to uncover the internally uneven development of urbanization across different built environments (LCZ 1–10) within the cities. For example, as the population growth at city center tended to stabilize, some industrial and suburb areas may experience more significant population growth. As we can see from Fig. 8, the maximum inter-annual change of population (ΔPD_max_) occurred in LCZ 10. For Zhongshan, the corresponding year of ΔPDmax of LCZ 10 was 1–2 years later than the rest of LCZs, which means heavy industrialization in Zhongshan still showed vivid development in the year of 2020 despite the general population growth of the city tended to slow down in 2008.

## Limitations

This study also has a few limitations that may influence accuracy of the results. LCZ heavily relies on remote sensing image observation datasets that capture surface features when simulating urban–rural gradients and further elucidating population distribution and change characteristics within the GBA. Therefore, the accuracy of data set acquisition and calibration plays a critical role in determining the effectiveness of LCZ applications. Additionally, the farming method on agricultural land at the urban built area’s edge will influence the determination of the research scope of the urban built area. Moreover, it has yet to be assessed whether the absence of population data for Macao will have a certain impact on the temporal and spatial changes in the overall population of the GBA.

## Conclusion and future work

In this study, local climate zone (LCZ) was used to analyze the spatio-temporal changes of population density in the Guangdong-Hong Kong-Macao Greater Bay Area (GBA). The growth of populations was evaluated across urban–rural gradients and years (2000–2020). We found that using LCZ for analyzing the spatio-temporal distribution dynamics of population within metropolitan areas offers distinct advantages and implications, compared to conventional studies reliant on impervious surfaces and land cover classification. From 2000 to 2020, the population dynamics in the GBA exhibited variations along urban–rural gradients and among different LCZ types. During this period, substantial population growth was observed in urban core areas, while suburban and rural areas experienced noticeable declines. Urban core emerged as population centers, with a discernible downward trend in population density along the urban–rural gradients. Moreover, the spatial distribution of population also exhibited notable differences across different LCZ types. Areas characterized by compact high-rise (LCZ 1) types, indicative of urban cores, showed the highest population density and greater concentration compared to other areas. Despite continuous population growth, the growth rate has been declining. Population density demonstrated an upward trajectory from 2000 to 2020, with the rate of change accelerating initially and then gradually slowing down. The interannual growth rate reached its peak in 2010 and has since declined.

Based on the research presented in this paper, future studies can further analyze the driving factors behind population changes in the GBA and explore changes in population structure, such as age and gender, as well as migration patterns. Additionally, when using LCZ to establish the urban–rural gradient, it should be complemented with high-resolution, long-term remote sensing imagery and population data to analyze the long-term, large-scale spatio-temporal changes to uncover the dynamics of national population change. Moreover, when applying LCZ, careful consideration should be given to the presence of distinctly heterogeneous urban structures in the study area, which can enhance the ability to sample urban surface features and improve the accuracy of employing LCZ for the analysis of rural–urban gradient expansion and spatio-temporal dynamics.

### Supplementary Information

Supplementary Information.

## Data Availability

Data is provided within the manuscript and supplementary information files.
