# Supplementary material for: Assessing spatiotemporal population density dynamics from 2000 to 2020 in megacities using urban and rural morphologies
Source: Sci Rep. 2024 Jun 19;14:14166. doi: 10.1038/s41598-024-63311-5 (PMC11187102; doi:10.1038/s41598-024-63311-5)
Supplement: Supplementary file 1 — Supplementary Information. [file 41598_2024_63311_MOESM1_ESM.docx]

Supplementary Materials for

**Title: Assessing spatiotemporal population density dynamics from 2000 to 2020 in megacities using urban and rural morphologies**

***Table S1***. Surface properties of local climate zones (LCZs) simplified from Stewart & Oke (2012).

| LCZ type | Built and land cover types | Anthropogenic  heat flux density^f^ | Aspect  ratio^a^ | Sky view  factor^b^ | Building  surface  fraction^c^ | Impervious  surface  fraction^d^ | Height of  roughness  elements^e^ |
| --- | --- | --- | --- | --- | --- | --- | --- |
| 1 | Compact high-rise | 50–300 | >2 | 0.2–0.4 | 40–60 | 40–60 | >25 |
| 2 | Compact mid-rise | <75 | 0.75–1.5 | 0.3–0.6 | 40–70 | 30–50 | 8–20 |
| 3 | Compact low-rise | <75 | 0.75–1.5 | 0.2–0.6 | 40–70 | 20–40 | 3–8 |
| 4 | Open high-rise | <50 | 0.75–1.25 | 0.5–0.7 | 20–40 | 30–40 | >25 |
| 5 | Open mid-rise | <25 | 0.3–0.75 | 0.5–0.8 | 20–40 | 30–50 | 8–20 |
| 6 | Open low-rise | <25 | 0.3–0.75 | 0.6–0.9 | 20–40 | 20–40 | 3–8 |
| 7 | Lightweight low-rise | <35 | 1–2 | 0.2– 0.5 | 60–90 | <10 | 2–4 |
| 8 | Large low-rise | <50 | 0.1–0.3 | >0.7 | 30–50 | 40–50 | 3–10 |
| 9 | Sparsely built | <10 | 0.1–0.25 | >0.8 | 10–20 | <20 | 3–8 |
| 10 | Heavy industry | >300 | 0.2–0.5 | 0.6–0.9 | 20–30 | 20–40 | 5–15 |
| A | Dense trees | 0 | >1 | <0.4 | <10 | <10 | 3–30 |
| B | Scattered trees | 0 | 0.25–0.75 | 0.5–0.8 | <10 | <10 | 3–15 |
| C | Bush, scrub | 0 | 0.25–1.0 | >0.9 | <10 | <10 | <2 |
| D | Low plants | 0 | <0.1 | >0.9 | <10 | <10 | <1 |
| E | Bare rock or paved | 0 | <0.1 | >0.9 | <10 | >90 | <0.25 |
| F | Bare soil or sand | 0 | <0.1 | >0.9 | <10 | <10 | <0.25 |
| G | Water | 0 | <0.1 | >0.9 | <10 | <10 | — |
| H | Wetlands | 0 | <0.1 | >0.9 | <10 | <10 | — |

^a^Mean height-to-width ratio of street canyons (LCZs 1–7), building spacing (LCZs 8–10), and tree spacing (LCZs A–G).

^b^Ratio of the amount of sky hemisphere visible from ground level to that for an unobstructed hemisphere.

^c^Proportion of ground surface with building cover (%).

^d^Proportion of ground surface with impervious cover (rock, paved) (%).

^e^Geometric average of building heights (LCZs 1–10) and tree/plant heights (LCZs A–F) (m).

^f^Mean annual anthropogenic heat flux density (Wm−2) at the local scale. Varies significantly with latitude, season, and population density.

^g^Wetlands is an additional LCZ type that adapted the land surface properties of coastal cities in Great Bay Area.

***Table S2***. Confusion matrix of the LCZ map of 1999 in the GBA region, obtained from Xie et al., (2022).

| LCZ | 1 | 2 | 3 | 4 | 5 | 6 | 7 | 8 | 9 | 10 | A | B | C | D | | E | F | G | H | No. Classified Pixels | User Accuracy |
| --- | --- | --- | --- | --- | --- | --- | --- | --- | --- | --- | --- | --- | --- | --- | --- | --- | --- | --- | --- | --- | --- |
| 1 | 247 | 40 | 58 | 56 | 18 | 17 | 9 | 2 | 1 | 23 | 0 | 4 | 1 | 23 | | 15 | 4 | 8 | 0 | 526 | 46.7% |
| 2 | 19 | 241 | 107 | 46 | 11 | 38 | 6 | 3 | 0 | 0 | 4 | 8 | 8 | 8 | | 4 | 0 | 0 | 0 | 503 | 47.9% |
| 3 | 1 | 10 | 891 | 7 | 9 | 4 | 22 | 10 | 3 | 24 | 0 | 1 | 2 | 1 | | 3 | 0 | 0 | 0 | 988 | 90.2% |
| 4 | 5 | 13 | 23 | 611 | 19 | 40 | 4 | 3 | 0 | 23 | 16 | 6 | 1 | 51 | | 26 | 0 | 18 | 3 | 862 | 70.9% |
| 5 | 4 | 4 | 15 | 13 | 340 | 17 | 0 | 0 | 5 | 0 | 21 | 3 | 0 | 14 | | 2 | 1 | 20 | 0 | 459 | 74.1% |
| 6 | 0 | 12 | 12 | 53 | 0 | 1005 | 0 | 13 | 0 | 1 | 2 | 6 | 3 | 0 | | 0 | 0 | 1 | 0 | 1108 | 90.7% |
| 7 | 2 | 20 | 31 | 4 | 3 | 1 | 474 | 0 | 0 | 11 | 0 | 2 | 0 | 13 | | 9 | 0 | 11 | 0 | 581 | 81.6% |
| 8 | 0 | 6 | 0 | 2 | 0 | 0 | 0 | 741 | 0 | 9 | 0 | 0 | 0 | 29 | | 18 | 0 | 6 | 0 | 811 | 91.4% |
| 9 | 0 | 0 | 0 | 0 | 0 | 11 | 0 | 0 | 173 | 0 | 7 | 6 | 8 | 0 | | 0 | 2 | 0 | 0 | 207 | 83.6% |
| 10 | 5 | 13 | 15 | 13 | 0 | 0 | 14 | 4 | 3 | 204 | 0 | 1 | 11 | 0 | | 23 | 4 | 52 | 2 | 364 | 56.0% |
| A | 0 | 0 | 1 | 0 | 0 | 4 | 0 | 0 | 0 | 0 | 1900 | 30 | 23 | 6 | | 0 | 5 | 0 | 0 | 1969 | 96.5% |
| B | 2 | 0 | 1 | 3 | 0 | 7 | 0 | 0 | 1 | 0 | 29 | 337 | 34 | 11 | | 0 | 2 | 31 | 0 | 458 | 73.6% |
| C | 2 | 0 | 5 | 5 | 0 | 11 | 2 | 0 | 0 | 1 | 204 | 337 | 504 | 20 | | 8 | 15 | 7 | 0 | 1121 | 45.0% |
| D | 4 | 0 | 0 | 25 | 0 | 8 | 4 | 9 | 0 | 2 | 24 | 131 | 300 | 1355 | | 31 | 2 | 77 | 3 | 1975 | 68.6% |
| E | 1 | 0 | 6 | 6 | 5 | 5 | 15 | 0 | 5 | 93 | 0 | 10 | 13 | 76 | | 165 | 1 | 68 | 1 | 470 | 35.1% |
| F | 4 | 4 | 10 | 23 | 0 | 28 | 1 | 33 | 0 | 7 | 16 | 15 | 9 | 126 | | 45 | 6 | 19 | 0 | 346 | 17.0% |
| G | 0 | 3 | 3 | 0 | 1 | 2 | 8 | 1 | 0 | 157 | 3 | 3 | 1 | 140 | | 3400 | 3700 | 113192 | 79 | 120693 | 93.8% |
| H | 0 | 0 | 0 | 0 | 0 | 0 | 2 | 0 | 0 | 0 | 0 | 0 | 0 | 100 | | 19 | 8 | 13 | 10 | 152 | 6.7% |
| No. Ground Truth Pixels | 296 | 366 | 1178 | 867 | 406 | 1198 | 561 | 819 | 191 | 555 | 2226 | 900 | 918 | 1973 | | 3768 | 3750 | 113523 | 98 | 133593 |  |
| Producer Accuracy | 83.5% | 65.9% | 75.6% | 70.5% | 83.7% | 83.9% | 84.5% | 90.5% | 90.6% | 36.8% | 85.4% | 37.4% | 54.9% | 68.7% | | 4.4% | 20.0% | 99.7% | 10.2% |  |  |
| Overall Accuracy | 91.6% |  | | **Built Accuracy** | | 71.1% |  | | **Land Coverings Accuracy** | | 92.0% |  | | |  | | | | | | |
| Kappa | 0.638 |  | | | | | | | | | |  | | |  |  |  |  |  |  |  |

***Table S3***. Confusion matrix of the LCZ map of 2009 in the GBA region, obtained from Xie et al., (2022).

| LCZ | 1 | 2 | 3 | 4 | 5 | 6 | 7 | 8 | 9 | 10 | A | B | C | D | | E | F | G | H | No. Classified Pixels | User Accuracy |
| --- | --- | --- | --- | --- | --- | --- | --- | --- | --- | --- | --- | --- | --- | --- | --- | --- | --- | --- | --- | --- | --- |
| 1 | 276 | 54 | 38 | 47 | 3 | 3 | 5 | 18 | 1 | 4 | 0 | 2 | 1 | 2 | | 7 | 1 | 0 | 0 | 462 | 59.7% |
| 2 | 9 | 293 | 32 | 6 | 6 | 2 | 2 | 0 | 0 | 0 | 0 | 1 | 0 | 9 | | 0 | 0 | 1 | 7 | 368 | 79.6% |
| 3 | 10 | 59 | 499 | 26 | 6 | 3 | 18 | 2 | 3 | 12 | 0 | 1 | 1 | 0 | | 5 | 2 | 0 | 1 | 648 | 77.0% |
| 4 | 58 | 18 | 31 | 643 | 31 | 11 | 6 | 9 | 0 | 4 | 17 | 9 | 1 | 0 | | 7 | 4 | 1 | 0 | 850 | 75.7% |
| 5 | 46 | 53 | 11 | 64 | 309 | 11 | 3 | 0 | 9 | 11 | 25 | 8 | 1 | 9 | | 9 | 1 | 0 | 13 | 583 | 53.0% |
| 6 | 10 | 9 | 15 | 27 | 6 | 645 | 9 | 4 | 9 | 18 | 21 | 4 | 6 | 2 | | 0 | 4 | 1 | 0 | 790 | 81.7% |
| 7 | 0 | 8 | 15 | 2 | 1 | 1 | 226 | 0 | 0 | 2 | 0 | 1 | 1 | 3 | | 2 | 0 | 2 | 1 | 265 | 85.3% |
| 8 | 6 | 7 | 12 | 8 | 0 | 3 | 0 | 497 | 0 | 59 | 0 | 0 | 1 | 1 | | 21 | 4 | 1 | 0 | 620 | 80.2% |
| 9 | 0 | 3 | 0 | 2 | 2 | 4 | 2 | 1 | 170 | 4 | 17 | 3 | 9 | 5 | | 8 | 9 | 0 | 0 | 239 | 71.1% |
| 10 | 25 | 22 | 28 | 13 | 5 | 33 | 11 | 32 | 7 | 289 | 2 | 3 | 31 | 18 | | 48 | 12 | 0 | 15 | 594 | 48.7% |
| A | 0 | 1 | 0 | 10 | 3 | 3 | 1 | 0 | 6 | 0 | 5925 | 71 | 25 | 4 | | 0 | 5 | 2 | 3 | 6059 | 97.8% |
| B | 15 | 0 | 0 | 42 | 0 | 3 | 0 | 0 | 0 | 0 | 152 | 190 | 126 | 5 | | 1 | 12 | 3 | 1 | 550 | 34.6% |
| C | 9 | 5 | 4 | 27 | 0 | 21 | 10 | 1 | 5 | 4 | 59 | 60 | 464 | 12 | | 3 | 88 | 4 | 13 | 789 | 58.8% |
| D | 2 | 2 | 0 | 13 | 0 | 0 | 3 | 3 | 8 | 0 | 141 | 44 | 35 | 915 | | 8 | 15 | 19 | 30 | 1238 | 73.9% |
| E | 16 | 13 | 19 | 13 | 1 | 5 | 22 | 84 | 1 | 46 | 3 | 7 | 17 | 344 | | 368 | 24 | 245 | 296 | 1524 | 24.2% |
| F | 22 | 7 | 13 | 19 | 5 | 10 | 9 | 17 | 5 | 37 | 54 | 21 | 78 | 55 | | 21 | 35 | 11 | 5 | 424 | 8.3% |
| G | 0 | 1 | 0 | 15 | 0 | 6 | 11 | 3 | 0 | 13 | 34 | 2 | 1 | 25 | | 13411 | 10 | 43495 | 135 | 57162 | 76.1% |
| H | 0 | 2 | 2 | 11 | 1 | 2 | 13 | 0 | 2 | 1 | 11 | 3 | 1 | 33 | | 5 | 19 | 129 | 2407 | 2642 | 91.1% |
| No. Ground Truth Pixels | 504 | 557 | 719 | 988 | 379 | 766 | 351 | 671 | 226 | 504 | 6461 | 430 | 799 | 1442 | | 13924 | 245 | 43914 | 2927 | 75807 |  |
| Producer Accuracy | 54.8% | 52.6% | 69.4% | 65.1% | 81.5% | 84.2% | 64.4% | 74.1% | 75.2% | 57.3% | 91.7% | 44.2% | 58.1% | 63.5% | | 2.6% | 14.3% | 99.1% | 82.2% |  |  |
| Overall Accuracy | 76.0% |  | | **Built Accuracy** | | 63.4% |  | | **Land Coverings Accuracy** | | 76.0% |  | | |  | | | | | | |
| Kappa | 0.566 |  | | | | | | | | | |  | | |  |  |  |  |  |  |  |

***Table S4***. Confusion matrix of the LCZ map of 2019 in the GBA region, obtained from Xie et al., (2022).

| LCZ | 1 | 2 | 3 | 4 | 5 | 6 | 7 | 8 | 9 | 10 | A | B | C | D | | E | F | G | H | No. Classified Pixels | User Accuracy |
| --- | --- | --- | --- | --- | --- | --- | --- | --- | --- | --- | --- | --- | --- | --- | --- | --- | --- | --- | --- | --- | --- |
| 1 | 359 | 82 | 19 | 87 | 10 | 5 | 4 | 2 | 0 | 6 | 0 | 1 | 0 | 0 | | 3 | 0 | 1 | 0 | 579 | 62.0% |
| 2 | 45 | 351 | 32 | 15 | 5 | 2 | 1 | 1 | 0 | 8 | 0 | 1 | 0 | 0 | | 0 | 0 | 0 | 0 | 461 | 76.1% |
| 3 | 28 | 66 | 1181 | 6 | 4 | 12 | 21 | 13 | 0 | 20 | 0 | 1 | 3 | 6 | | 4 | 1 | 0 | 0 | 1366 | 86.5% |
| 4 | 127 | 35 | 35 | 958 | 105 | 77 | 3 | 2 | 1 | 16 | 34 | 24 | 8 | 2 | | 9 | 2 | 3 | 0 | 1441 | 66.5% |
| 5 | 15 | 11 | 27 | 48 | 286 | 21 | 9 | 0 | 4 | 5 | 10 | 0 | 2 | 55 | | 2 | 1 | 0 | 8 | 504 | 56.8% |
| 6 | 7 | 6 | 22 | 47 | 13 | 780 | 32 | 9 | 1 | 13 | 6 | 17 | 22 | 108 | | 7 | 3 | 0 | 15 | 1108 | 70.4% |
| 7 | 8 | 16 | 138 | 8 | 6 | 7 | 476 | 5 | 2 | 0 | 0 | 1 | 1 | 14 | | 0 | 0 | 1 | 1 | 684 | 69.6% |
| 8 | 10 | 30 | 80 | 8 | 3 | 2 | 2 | 677 | 0 | 36 | 0 | 2 | 0 | 7 | | 51 | 19 | 0 | 0 | 927 | 73.0% |
| 9 | 0 | 1 | 8 | 3 | 4 | 11 | 3 | 1 | 119 | 5 | 18 | 16 | 20 | 66 | | 7 | 13 | 1 | 3 | 299 | 39.8% |
| 10 | 16 | 34 | 17 | 26 | 1 | 7 | 25 | 23 | 0 | 359 | 0 | 2 | 2 | 11 | | 18 | 1 | 2 | 0 | 544 | 66.0% |
| A | 0 | 0 | 0 | 0 | 4 | 6 | 0 | 0 | 12 | 0 | 6056 | 137 | 109 | 3 | | 0 | 1 | 1 | 0 | 6329 | 95.7% |
| B | 0 | 0 | 3 | 1 | 0 | 9 | 0 | 1 | 2 | 0 | 95 | 242 | 68 | 14 | | 0 | 0 | 1 | 0 | 436 | 55.5% |
| C | 0 | 2 | 0 | 4 | 5 | 15 | 5 | 0 | 8 | 14 | 31 | 90 | 220 | 28 | | 3 | 24 | 0 | 5 | 454 | 48.5% |
| D | 0 | 2 | 4 | 2 | 0 | 3 | 0 | 6 | 3 | 7 | 165 | 104 | 71 | 2474 | | 1 | 7 | 3 | 107 | 2959 | 83.6% |
| E | 4 | 6 | 19 | 2 | 1 | 1 | 4 | 25 | 0 | 24 | 0 | 1 | 0 | 27 | | 264 | 18 | 1 | 41 | 438 | 60.3% |
| F | 3 | 1 | 0 | 1 | 1 | 4 | 2 | 2 | 2 | 0 | 2 | 11 | 27 | 4 | | 13 | 37 | 0 | 1 | 111 | 33.3% |
| G | 1 | 3 | 2 | 5 | 0 | 0 | 1 | 1 | 0 | 0 | 5 | 1 | 0 | 78 | | 2 | 2 | 42828 | 133 | 43062 | 99.5% |
| H | 0 | 0 | 0 | 1 | 0 | 0 | 2 | 0 | 2 | 0 | 54 | 6 | 12 | 97 | | 3 | 1 | 190 | 744 | 1112 | 66.9% |
| No. Ground Truth Pixels | 623 | 646 | 1587 | 1222 | 448 | 962 | 590 | 768 | 156 | 513 | 6476 | 657 | 565 | 2994 | | 387 | 130 | 43032 | 1058 | 62814 |  |
| Producer Accuracy | 57.6% | 54.3% | 74.4% | 78.4% | 63.8% | 81.1% | 80.7% | 88.2% | 76.3% | 70.0% | 93.5% | 36.8% | 38.9% | 82.6% | | 68.2% | 28.5% | 99.5% | 70.3% |  |  |
| Overall Accuracy | 93.0% |  | | **Built Accuracy** | | 68.04% |  | | **Land Coverings Accuracy** | | 95.2% |  | | |  | | | | | | |
| Kappa | 0.864 |  | | | | | | | | | |  | | |  |  |  |  |  |  |  |


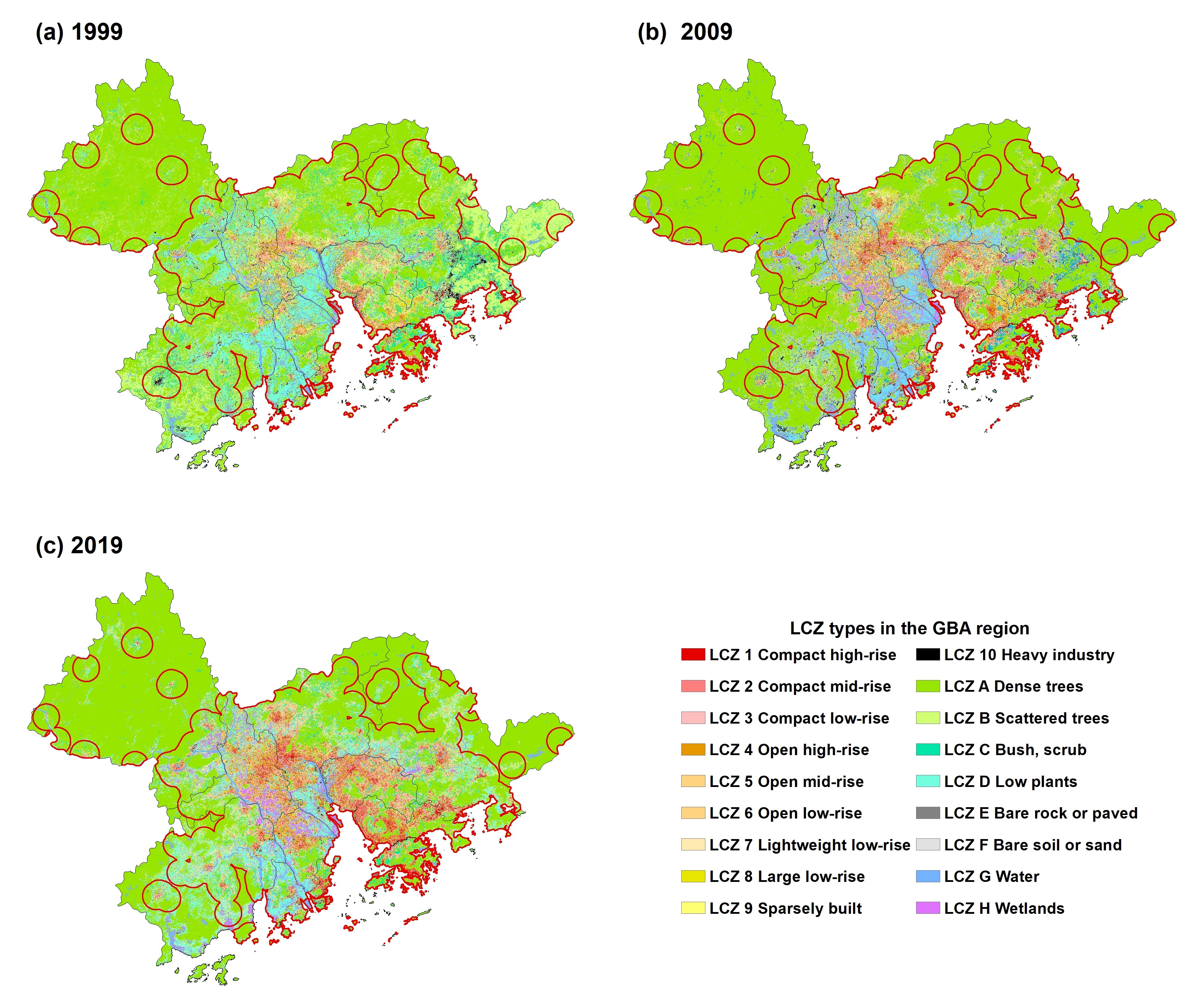


***Figure S1***. Spatial patterns of local climate zone (LCZ) in 1999 (a), 2009 (b), and 2019 (c) in the GBA region. LCZ maps obtained from Xie et al., (2022), and the red boundaries present a distance gradient that built LCZ areas large than 1% in 2019. Figure mapping visualizations were performed in ArcGIS (v10.8, ESRI, USA).


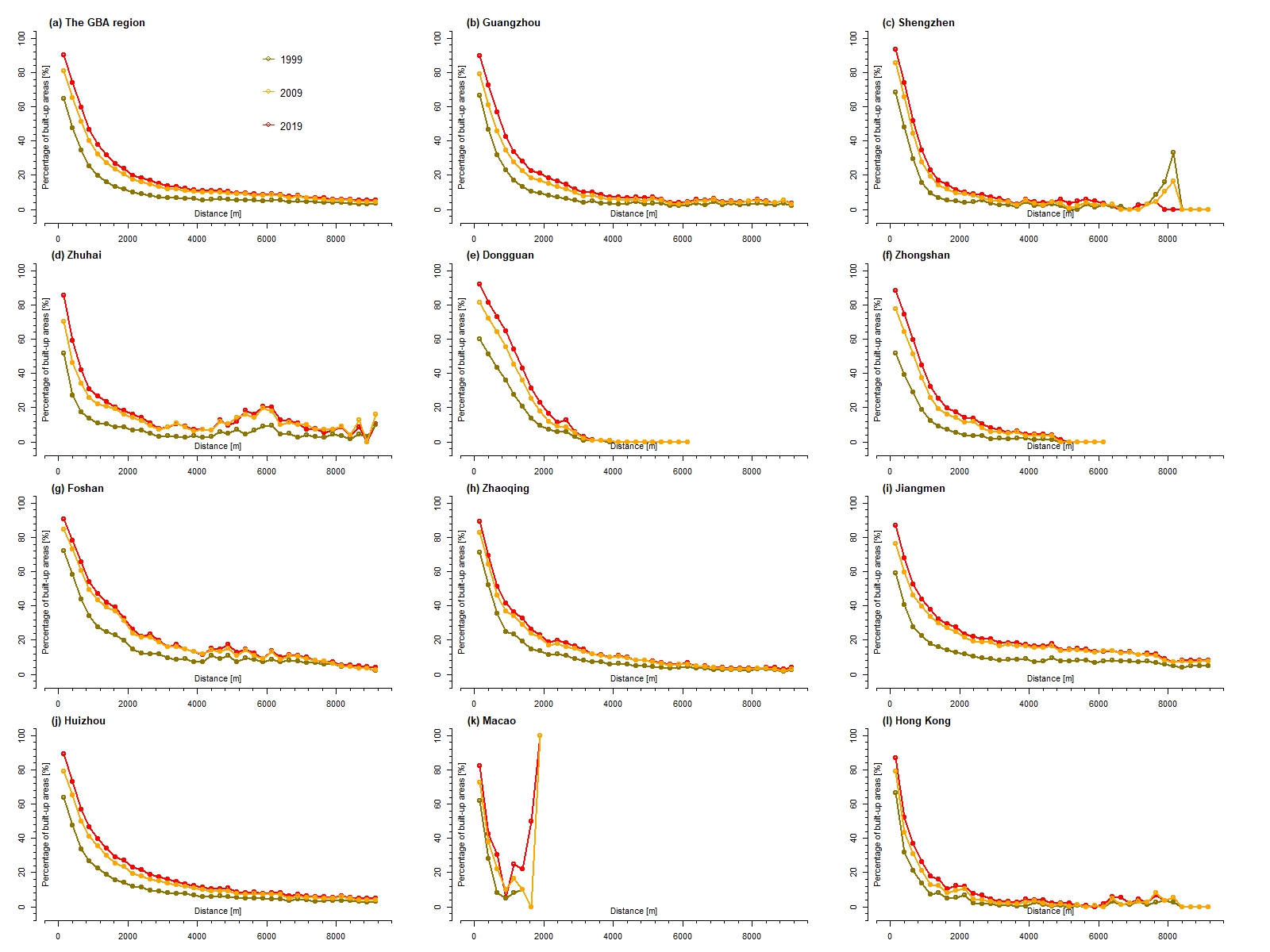


***Figure S2***. Urban-rural densities of built areas (LCZ 1–10) for the GBA region and each city in 1999, 2009, and 2019 obtained from Xie et al., (2022).


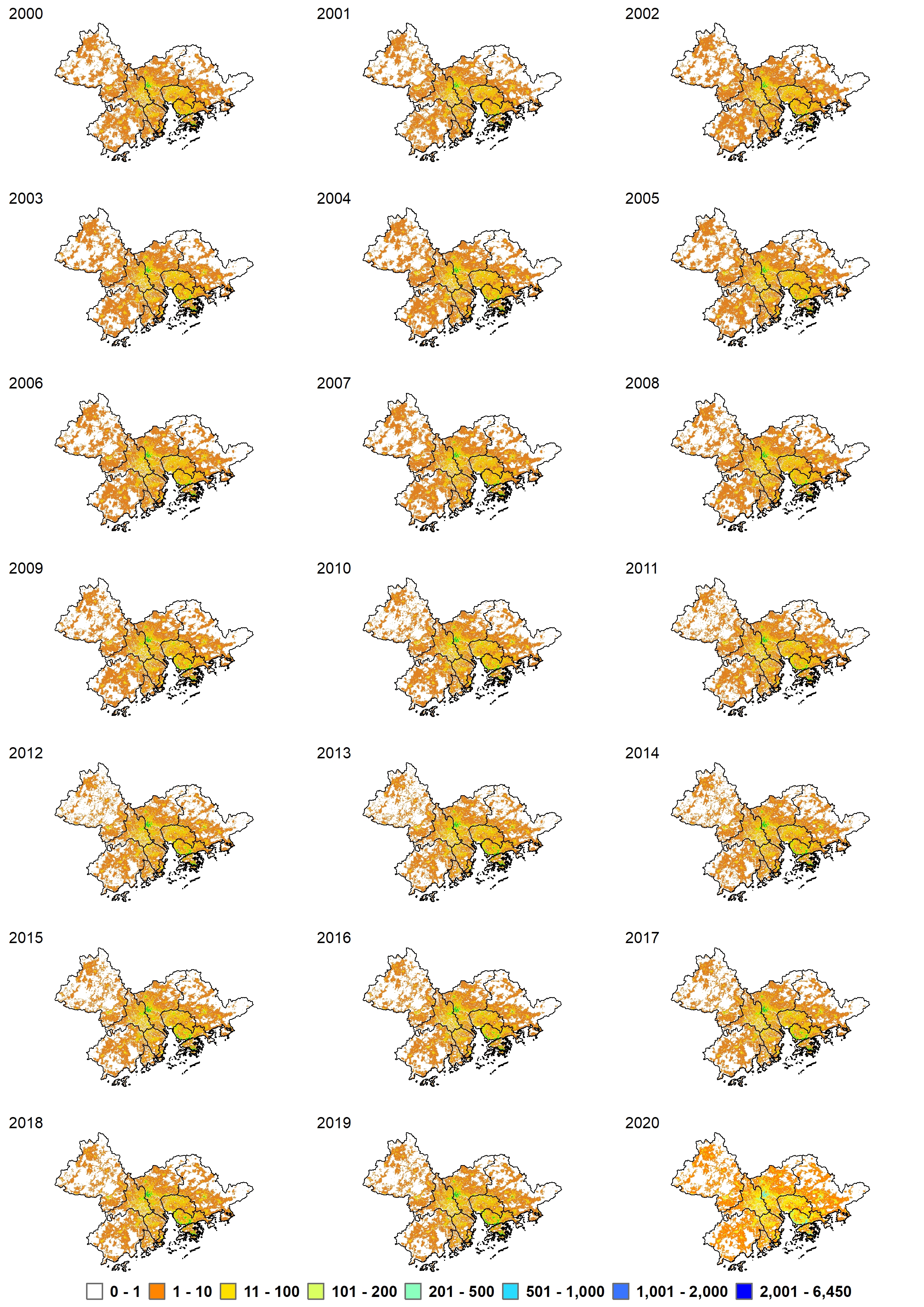


***Figure S3.*** The spatial distribution of population between 2000–2020 with cities in the Guangdong-Hong Kong-Macao Greater Bay Area (GBA). Figure mapping visualizations were performed in ArcGIS (v10.8, ESRI, USA).


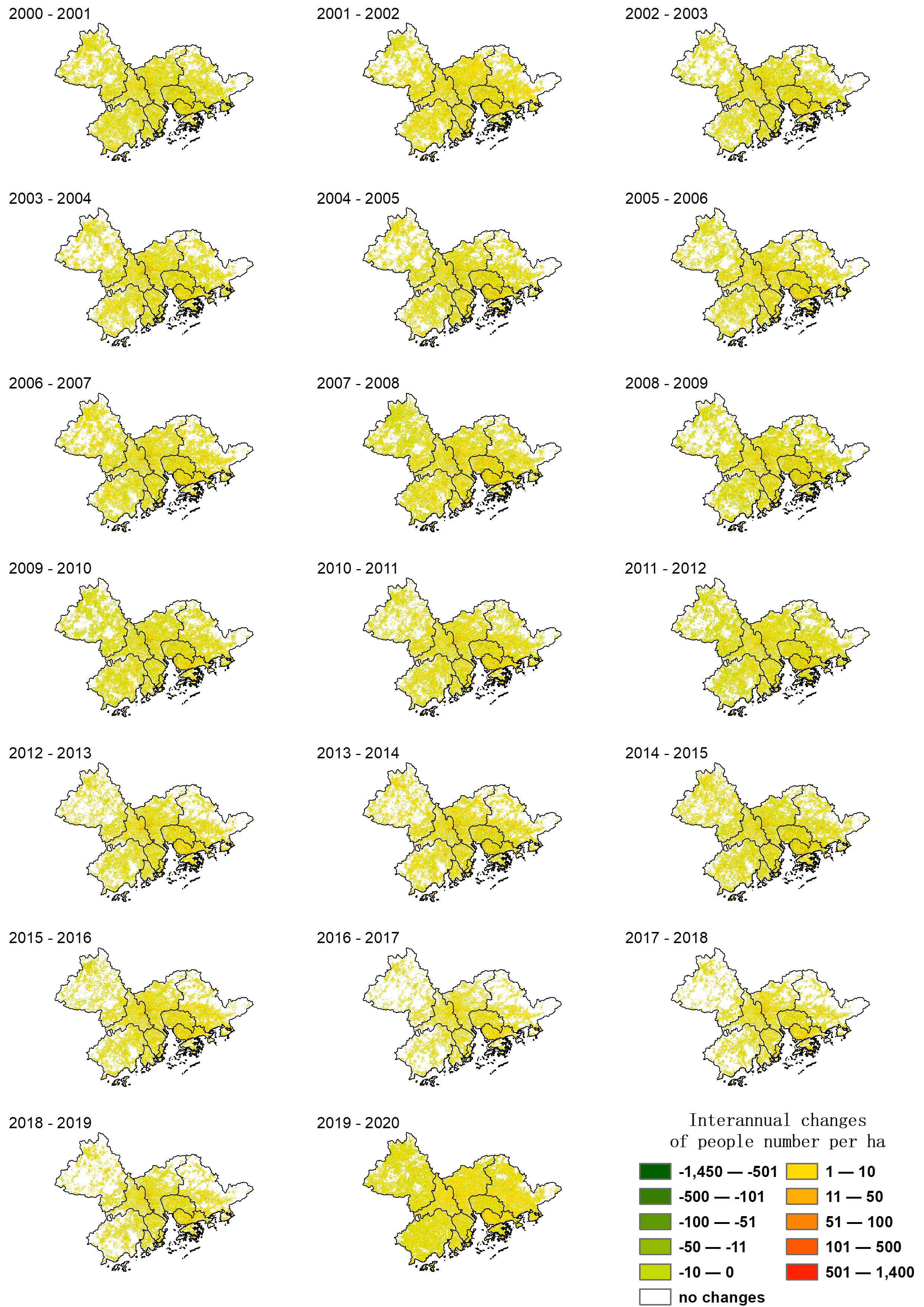


***Figure S4.*** The spatial distribution of interannual changes of population between 2000–2020 with cities in the Guangdong-Hong Kong-Macao Greater Bay Area (GBA). Figure mapping visualizations were performed in ArcGIS (v10.8, ESRI, USA).


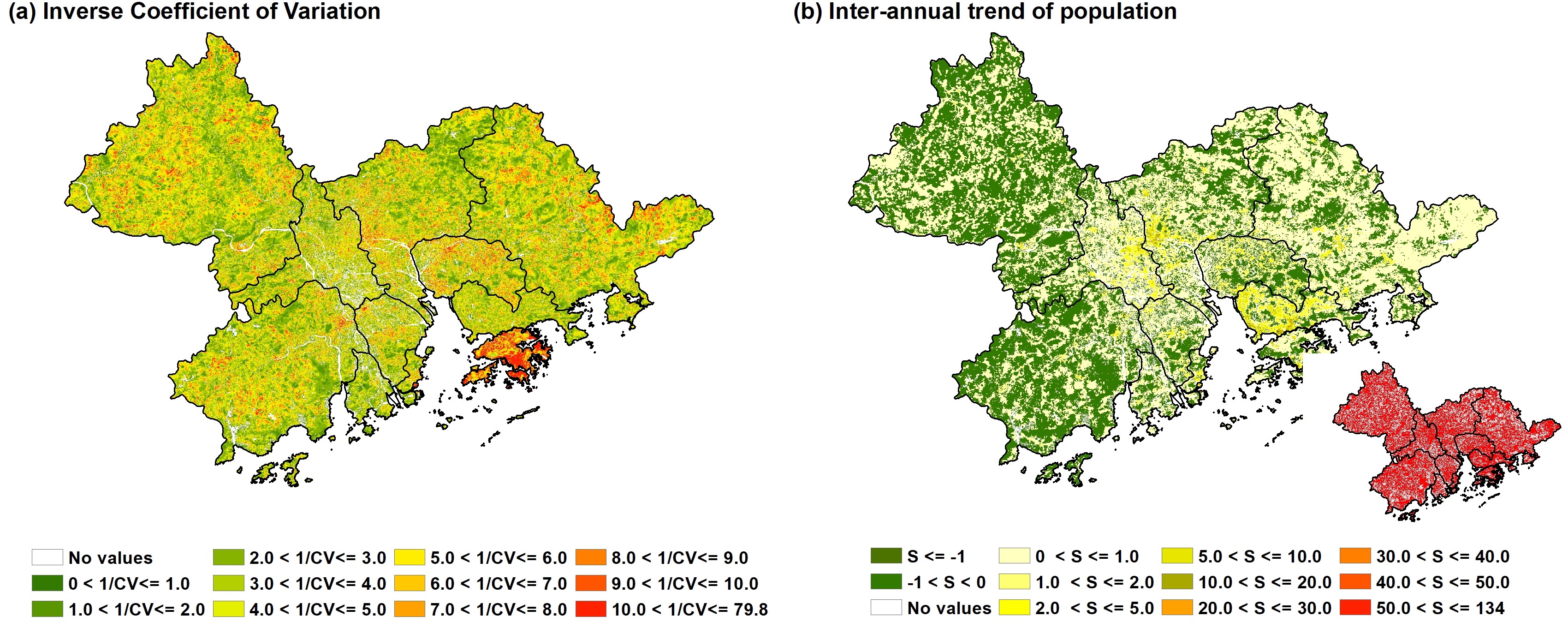


***Figure S5.*** Inverse coefficient of variation (CV^-1^) (a) and interannual trend of populations and areas with significant trend (red patterns) (b) for the GBA region between 2000–2020. Figure mapping visualizations were performed in ArcGIS (v10.8, ESRI, USA).


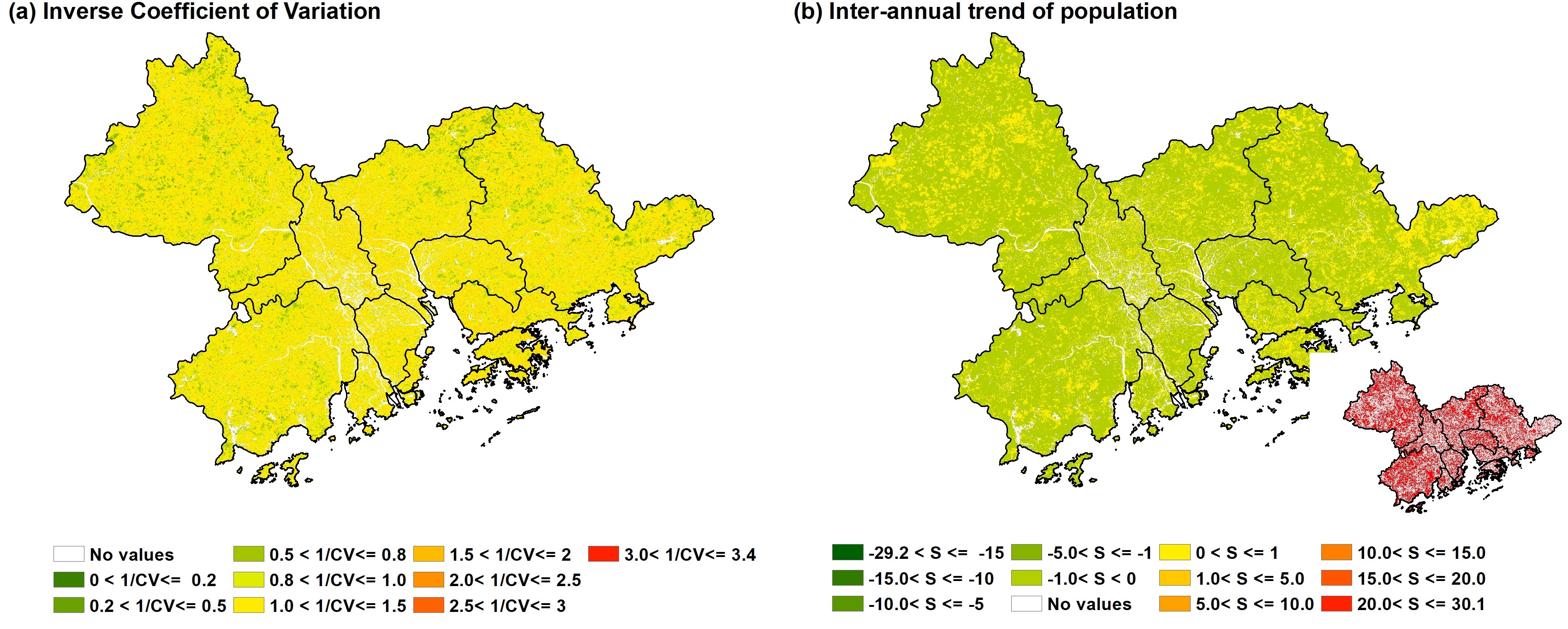


***Figure S6.*** Inverse coefficient of variation (CV^-1^) (a) and trend of abstract interannual difference (Δ) of populations and areas with significant trend (red patterns) (b) for the GBA region between 2000–2020. Figure mapping visualizations were performed in ArcGIS (v10.8, ESRI, USA).


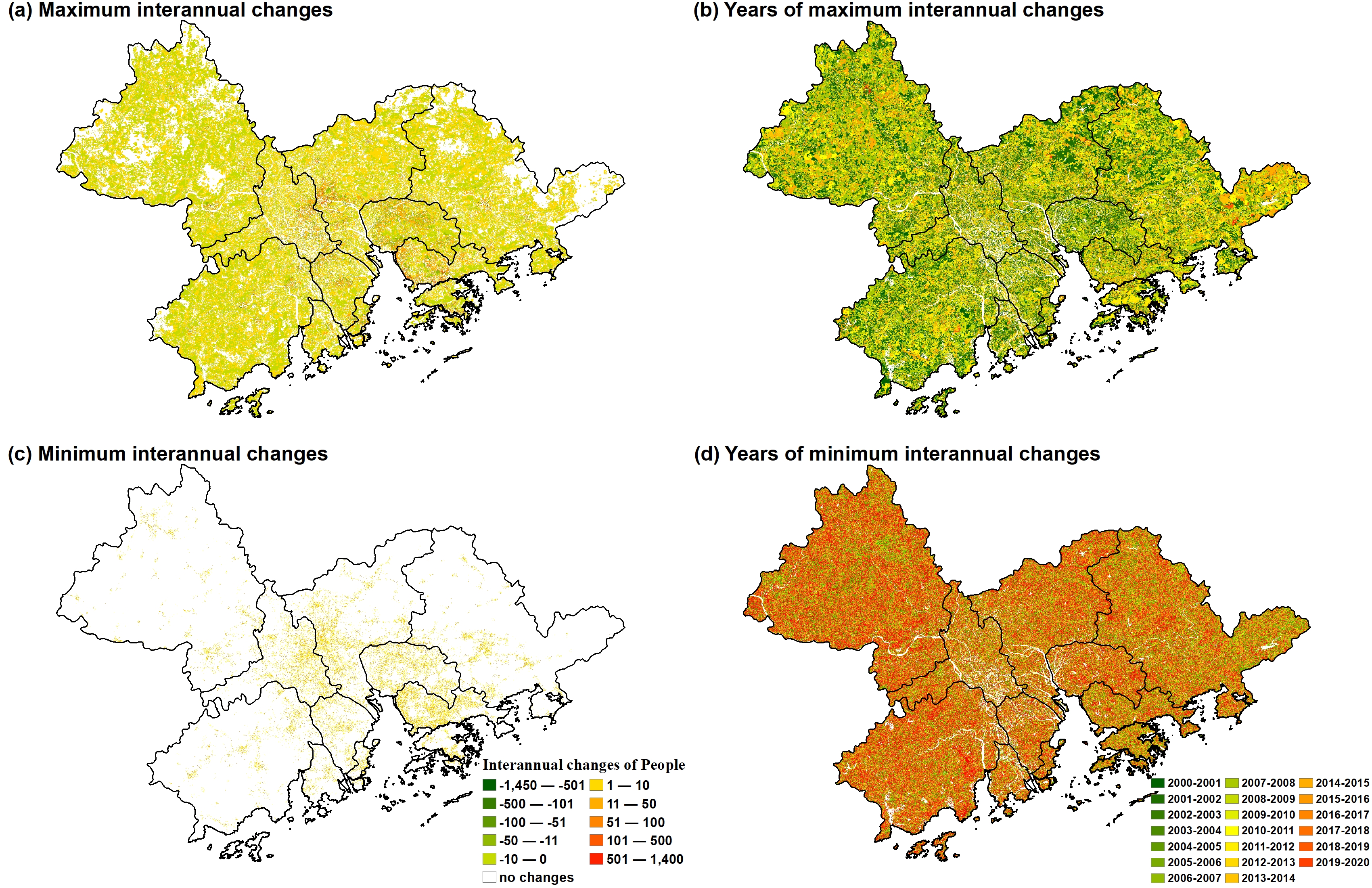


***Figure S7.*** Maximum (a) and minmum (c) interannual changes of populations, and the corresponding years with maximum (b) and minmum (d) abstract interannual difference (Δ) of populations. Figure mapping visualizations were performed in ArcGIS (v10.8, ESRI, USA).

References:

Stewart, I. D., & Oke, T. R. (2012). Local climate zones for urban temperature studies. *Bulletin of the American Meteorological Society*. https://doi.org/10.1175/BAMS-D-11-00019.1
